# (Cost)-effectiveness and implementation of integrated community-based care for patients with severe mental illness: a study protocol

**DOI:** 10.1186/s12888-022-04346-8

**Published:** 2022-11-11

**Authors:** Anne Kleijburg, Ben Wijnen, Silvia M. A. A. Evers, Hans Kroon, Joran Lokkerbol

**Affiliations:** 1 Department of Health Services Research, CAPHRI Care and Public Health Research Institute, Maastricht University, Maastricht, The Netherlands; 2 Centre of Economic Evaluations & Machine Learning, Trimbos Institute, Netherlands Institute of Mental Health and Addiction, Utrecht, The Netherlands; 3 Department of Social and Behavioural Sciences, Tranzo Scientific Center for Care and Welfare, Tilburg University, Tilburg, Netherlands; 4 Department of Reintegration and Community Care, Trimbos Institute, Netherlands Institute of Mental Health and Addiction, Utrecht, The Netherlands

**Keywords:** Integrated care, Severe mental illness, Flexible assertive community treatment, Mental healthcare, Social services, Cost-effectiveness, Implementation

## Abstract

**Background:**

As severe mental illness (SMI) is associated with a high disease burden and persistent nature, patients with SMI are often subjected to long-term mental healthcare and are in need of additional social support services. Community-based care and support services are organized via different providers and institutions, which are often lacking structural communication, resulting in a fragmented approach. To improve the efficiency of care provision and optimize patient wellbeing, an integrated multi-agency approach to community-based mental health and social services has been developed and implemented.

**Aim:**

To present a research protocol describing the evaluation of flexible assertive community teams integrated with social services in terms of effectiveness, cost-effectiveness, and implementation.

**Methods/design:**

A quasi-experimental study will be conducted using prospective and retrospective observational data in patients with severe mental illness. Patients receiving care from three teams, consisting of flexible assertive community treatment and separately provided social support services (care as usual), will be compared to patients receiving care from two teams integrating these mental and social services into a single team. The study will consist of three parts: 1) an effectiveness evaluation, 2) a health-economic evaluation, and 3) a process implementation evaluation. To assess (cost-)effectiveness, both real-world aggregated and individual patient data will be collected using informed consent, and analysed using a longitudinal mixed model. The economic evaluation will consist of a cost-utility analysis and a cost-effectiveness analysis. For the process and implementation evaluation a mixed method design will be used to describe if the integrated teams have been implemented as planned, if its predefined goals are achieved, and what the experiences are of its team members.

**Discussion:**

The integration of health and social services is expected to allow for a more holistic and recovery oriented treatment approach, whilst improving the allocation of scarce resources. This study aims to identify and describe these effects using a mixed-method approach, and support decision-making in the structural implementation of integrating mental and social services.

## Background

Severe mental illness (SMI) is defined as the suffering from a psychiatric disorder with a duration of at least 2 years, which substantially interferes with multiple domains in life, thereby negatively impacting determinants of wellbeing such as educational attainment, work productivity, financial stability and housing, maintaining personal relations and life expectancy [1]. As such, SMI not only heavily impacts the quality of life of affected individuals, but also introduces substantial healthcare and societal costs [2, 3]. As a result, increasing consensus exists that recovery-oriented treatment of SMI requires coordinated long-term care that integrates not only mental healthcare but also other domains, such as the social or work domain [4]. In the Netherlands the prevalence of patients suffering from SMI is estimated at 1.7%, from which 43% continues to receive care for more than 6 years in a row [4, 7].

Both community-based mental and social services are available for patients with SMI in the Netherlands. Ambulatory mental healthcare services are provided by teams operating according to the Flexible Assertive Community Treatment (FACT) model, the Dutch version of Assertive Community Treatment (ACT). Basic principles of the ACT model recommend that assertive outreaching intensive care is provided by a multidisciplinary team, using a shared caseload and covering 24 hours a day [8]. In addition, the Dutch FACT model, implemented first in 2002, has added the ability to provide this care more flexibly, allowing treatment intensity and the degree of multidisciplinary involvement to vary according to the patient’s need [9]. If patients need a high treatment intensity (a varying subsample of approximately 15-20%) a shared caseload and assertive outreach approach may be adopted during which they are regularly discussed in the daily FACT team meetings and receive frequent home-visits and treatment sessions. Patients in a more stable phase (±80-85% of the caseload) can continue to be managed individually within the FACT team with 2-4 case manager visits a month. This ability to provide different levels of treatment intensity within a single team, and switch when necessary, improves treatment continuity and decreases drop-out rates. The multidisciplinary FACT team generally includes a psychiatrist, case managers/nurses, a psychologists, a peer support specialist and an employment specialist, usually with a staff:patient ratio of 1:20 [10]. The Centre for Certification of ACT and FACT certifies FACT teams within the Netherlands according to its fidelity handbook.

FACT teams in the Netherlands are usually financed by health insurers. However, in 2015 a distinction between treatment (financed by health insurers) and care and participation (financed by municipalities) was introduced, challenging the scope and integration of services within FACT. At that time new District Social Service Teams (DSST) were set up adjacent to FACT.

Services provided to community members by (or organized via) the DSST may include psychosocial support, household help, financial support, housing, day activity centers, employment and re-integration programs, and coaching. Given that patients suffering from SMI often also experience problems in these areas, services by these DSSTs are indicated in over half of the cases (based on unpublished results from a local business case in preparation of implementation). Though the expertise and variety of available social services significantly improved following the introduction of the DSST, a secondary effect was that services catering to the needs of SMI patients were now organized by multiple parties, requiring newfound consensus on how to organize local collaboration and responsibilities. To manage the opportunities and challenges that arose from this change in the landscape, FACT teams were requested to create a custom cross-domain approach by collaborating with local social services providers [11]. Experts in the field believe that in order to maximize recovery and participation, stimulate prevention of future deterioration, and minimize overall expenditures, ideally, treatment, care and support services must be coordinated efficiently amongst the different providers, with their intensities being dynamically up- or downscaled based on patient need [10]. However, despite continuous efforts and developments in the provision of this cross-domain integrated (FACT) care, communication is inconsistent across patients, and structural collaboration between community-based mental healthcare and municipal social support services remain suboptimal. As a result, this often translates to more responsibility (and confusion) for patients to manage their service agreements.

To better integrate these services, a two-year study has been initiated in the Dutch province of Friesland, implementing the FACT+DSS teams, an intersectoral and multi-agency approach. Here, social workers from the DSSTs integrate with the FACT teams, realizing direct communication between municipal and healthcare services. The rationale behind this approach is to improve conditions for both its health and social workers and patient population. For its team members, integration aims to promote the sharing of knowledge and expertise across the different specialties, shorten the communicative lines with other service providers, improve awareness of the available health and social services, and reduce time spent on administrative procedures. For its patient population, integration aims to create a higher quality experience in the provision of health and social services, including the accessibility of these services, allowing for more dynamic, responsive and personalized recovery plans, stimulating social participation and recovery. A part of this is, for example, that patients who refuse treatment by mental health services (care-avoiders) whilst still receiving social support can be included in the teams’ caseload by their social workers.

The study described in this protocol aims to evaluate the FACT+DSS teams in terms of effectiveness, cost-effectiveness, and its implementation process and outcomes, analyzing both quantitative and qualitative data generated throughout the study.

## Methods

This protocol describes three different evaluations, i.e. effect evaluation, economic evaluation and a process and implementation evaluation, each with their own research questions:

1. Effect evaluation
  1. What effect do the FACT+DSS teams have on the wellbeing of patients with SMI, looking at Health of the Nation Outcome Scores (HoNOS) and Quality of Life (QoL), when compared to care as usual (CAU)?
2. Economic evaluation
  Primary
  1. What is the cost-effectiveness of FACT+DSS teams compared to CAU, as considered from a societal perspective in the Netherlands (i.e. looking at both incremental costs per QALY gained and incremental costs per responder to treatment)?
  Secondary
  2) What is the estimated budget impact of adopting the FACT+DSS approach?
3. Process and implementation evaluation
  Primary
  1. Has the integration of community-based services by FACT teams and DSSTs been implemented as planned and are the study’s predefined goals obtained (e.g. stakeholder solidarity, model fidelity, cooperation)?
  2. How did members of the team experience the implementation process and how do they evaluate the care delivered by the FACT+DSS teams (e.g. added patient value, reduced administrative burden, increased direct care time)?
  Secondary
  1. What are lessons learned and recommendations for future implementation of the FACT+DSS teams and rollout in other regions/municipalities?
  2. What effect do the FACT+DSS teams have on the size and nature of the target group reached (e.g. changes in number of care avoiders, up/downscaling team load) and does this impact costs of care delivered?

### Study design

**Fig. 1:**
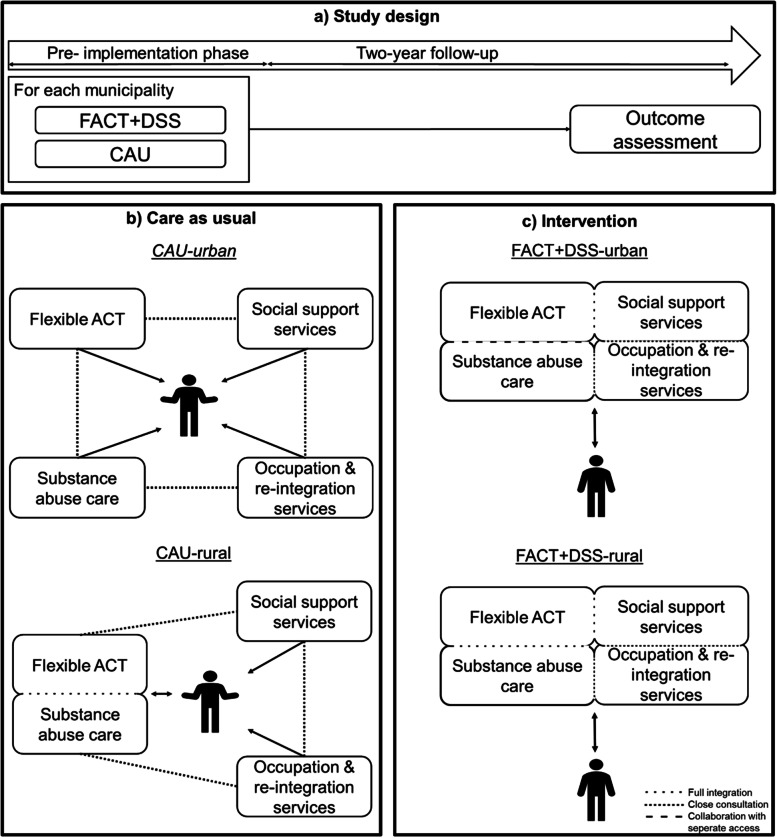
Schematic representation of (**a**) the study design in the evaluation of the FACT+DSS teams, and the health and social service provider network in the Netherlands illustrating (**b**) care as usual (CAU) where services are provided by separate organizations, and (**c**) after implementation of the integrated FACT+DSS teams. For both CAU and the FACT+DSS teams a distinction is made between the rural and urban area teams, where substance abuse is either integrated fully (rural) or by close collaboration with separate access. Occupational and reintegration services are accessible through a dedicated consultant

A longitudinal, quasi-experimental study design using both prospective and retrospective observational data is applied to compare the FACT+DSS teams with CAU, consisting of separate FACT and DSSTs. The teams have been implemented as part of a pragmatic study during which two FACT+DSS teams and three CAU teams are operational. The study phase during which the integration of services is implemented has a duration of 2 years after which the FACT+DSS teams are planned to continue to exist, replacing CAU within the region. Collection of observational client data for longitudinal analysis will revert around 1.5 years prior to the start of the study, up until the end of the study, resulting in a total of 3.5 years. Figure 1 shows a schematic representation of the design. Results of this study will be reported in line with the Strengthening the Reporting of Observational Studies in Epidemiology (STROBE) guidelines and the Dutch guidelines for economic evaluation [12, 13]. As the FACT+DSS teams have replaced CAU in the region, and our evaluations require no additional procedures for patients, this study has received the classification of a non-interventional study by the Medical Ethics Review Committee of the University Hospital Maastricht and Maastricht University (application no. 2021-2868), and was assessed for its local feasibility by the Scientific Committee of GGZ Friesland.

### Study population

The target population for this study consists of people receiving care from the FACT+DSS teams or the FACT and/or DSSTs separately (care as usual), providing care to FACT+DSS-eligible patients with severe mental illness. Patients must receive services from either the FACT+DSS or CAU teams which are active in the scope of this study. Patients are eligible to be included in the study if they are over 18 years old and are able to provide informed written consent. To assess the patients’ ability to provide informed consent and their ability to oversee the consequences, their psychiatrist will be asked to declare the patients’ current state of mental competence.

### Setting

FACT+DSS and CAU teams are active in two Dutch municipalities, where one is more urban (Leeuwarden, 526 inhabitants per KM^2^) and the other more rural (Súdwest-Fryslân, 172 inhabitants per KM^2^), providing diverse demographic and socio-economic characteristics to be considered throughout the comparative analyses [14]. In the urban and rural municipality three and two FACT teams are active, respectively, where each team is responsible for a specific sub-region within the municipality.

CAU consists of three FACT teams, of which two are active in the urban municipality of Leeuwarden (CAU-urban), and one is active in the rural municipality Súdwest-Fryslân (CAU-rural). For the FACT+DSS teams, again one is active in the urban area (FACT+DSS-urban) and one in the rural area (FACT+DSS-rural).

### Informed consent

All patients in the FACT+DSS CAU teams are asked if they are willing to provide informed consent for sharing personal information collected by different service providers, to be used in the effect and economic evaluation. All information regarding the study and participation is provided both in written and in verbal explanation by the patient’s case manager. When interested in participation, patients are guided through the full consent form and asked to provide consent for each service provider separately. To ensure all important elements of participation are discussed and fully understood, use is made of an informed consent checklist. The privacy of all participant data will be protected using coded and secured data storage, and anonymized data analysis. The participants will be made explicitly aware of their rights related to terminating participation in the study.

### Usual care

The integrated teams are compared to CAU, which here consists of mental care provided by a FACT team and social support services provided separately by the DSSTs. Here, the multidisciplinary FACT team operates according to the principles of the FACT fidelity scale as introduced earlier, where patients are assigned a case manager that is providing outreaching, psychiatric treatment and support. These outreaching services are provided to patients independent of their living situation, being either in their homes, on the street when homeless, or at supported housing facilities when requiring continuous supervision. Additionally, case managers are encouraged to coordinate multi-agency collaboration across involved care and service providers with periodic (but often sporadic) meetings [10]. The number and types of additional providers involved with a patient vary on individual needs, but besides the DSSTs frequent partners are local substance abuse care providers, occupational support services of the Dutch Employee Insurance Agency (EIA, in Dutch: UWV), supported living facility case managers, or other local (health or support) services providers available to patients with SMI that are out of scope for this evaluation.

The CAU-rural team includes specialists (physicians, nurses) from a separate substance abuse treatment centre, while in the CAU-urban teams addiction care is not integrated. This difference between CAU teams, and their place in the network of service providers surrounding patients with SMI is illustrated in Fig. 1b. On average, the CAU teams consist of a psychiatrist, a psychologist, 2-3 ambulatory psychiatric/addiction nurses, 2-3 social psychiatric nurses, an Individual Placement and Support counsellor (IPS), and a peer-support worker, with a total ranging from 8.5 to 10.5 FTE. All CAU teams have an estimated caseload ranging from 140 to 190 individuals, with a staff to patient ratio of 1:14 to 1:18.

These DSSTs are an initiative of Dutch municipalities to support its citizens in societal participation and self-management when needed. DSSTs provide and coordinate a wide variety of support services for issues related to e.g. housing, work and income, social contacts, family and child relations, and domestic violence. Disciplines active in the DSSTs generally include social workers, but also various specialized consultants, nurses, and volunteers. A case worker of the DSSTs (or Social Community Teams) provides patients with additional social support services if needed.

### FACT+DSS

The new FACT+DSS approach consists of the integration of regular FACT teams with social workers from the DSSTs, resulting in the formation of two FACT+DSS teams. These FACT+DSS teams consist of all members of the former FACT team and a minimum of two, but preferably more, DSST social workers. In addition to this, the implementation design describes that an occupational consultant of the Employee Insurance Agency is assigned to each team to stimulate employment reintegration, and each team is to have access to a substance abuse specialist from the local addiction care provider, either by integration in the team itself or by collaboration with separate access. Similar to CAU-rural, the FACT+DSS-rural team had an addiction specialist integrated in the team prior to implementation. The FACT+DSS-urban team collaborates with substance abuse specialists by separate access. This is also illustrated in Fig. 1c, which shows that patients are no longer approached by separate providers but rather by a multi-agency, cross-domain team. During the two-year implementation phase each team is supported by a coach to guide them through the process, support them in solving problems that may arise, and ensure communication to project leaders and higher management.

Compared to CAU, the FACT+DSS teams are expected to provide health and social services more efficiently for those who require both services by using an integrated, individualized treatment approach. Activities of the FACT+DSS teams are similar to that of the CAU teams and according to the FACT model guidelines. Novel aspects of their day-to-day work activities include the participation of the social workers in the daily morning meetings, collaboratively discussing client cases, creating cross-domain case management plans aimed at recovery, performing joint house visits, and documenting case updates in a single electronic patient file. Similar to the FACT case managers, social workers are (joint) case managers to a group of patients. These patients may be either introduced by them in the FACT+DSS caseload or patients adopted through standard routes who’s personal goals at that time are best aligned with the social workers expertise. An important characteristic of these integrated teams is that social workers in the FACT+DSS teams are authorized to grant patients their indication for support services immediately, significantly reducing waiting times for patients to start with the requested service.

Upon implementation, the FACT+DSS team size increases by the integration of social workers, creating an estimated team size of 11 to 14 FTE. Additionally, expectations are that the number of patients served will increase due to enrolment of DSST-clients that are expected to benefit from mental health services. Overall a staff:patient ratio of 1:14 to 1:17 is expected.

### Effect evaluation

To assess the effect of care delivered by FACT+DSS teams on patients, use will be made of several patient-related outcome measures. All outcome measures used in this observational effectiveness evaluation are derived from real-world data, collected as a result of the patients’ use of services and the organizations’ standard administrative practices by means of routine outcome monitoring (ROM).

Primary outcome measures:

1. Health of the Nation Outcome Scores (HoNOS). The HoNOS is a questionnaire evaluating mental and social functioning and is filled out yearly by treating physicians to monitor the patient’s progress [15].
2. Quality of life. As no generic quality of life instrument is included in the ROM data, a mapping study will be performed using the HoNOS and EuroQol 5 Dimension 3 level (EQ-5D-3L) utilities to obtain estimates of generic quality of life (note: this mapping is part of a separate study using data not collected as part of the study described here). To this extent, EQ-5D-3L scores will be converted to country-specific utility tariffs [16]. The EQ-5D-3L is a health-related questionnaire assessing five dimensions (mobility, self-care, usual activities, pain/discomfort, anxiety/depression) where each dimension can be scored using three levels (no problems, moderate problems, severe problems).

Secondary outcome measure:

1. Global Assessment of Functioning (GAF) scores. The GAF instrument is a generic measure used to score patients between 1 and 100 on their overall wellbeing [17. ]. The GAF was part of the Diagnostic and Statistical Manual of Mental Disorders Fourth Edition (DSM-IV) and is included in the Dutch system for declaring healthcare costs until 2022.

### Economic evaluation

An economic evaluation will be performed consisting of a cost-effectiveness analysis (CEA) and a cost-utility analysis (CUA), both considering a societal perspective. For the CEA, HoNOS scores will be dichotomised to calculate the cost per responder, where being a responder (yes/no) is determined using the methods by Jacobsen & Truax for calculating reliable and clinically relevant change from baseline to follow-up [18]. For the CUA, health outcomes will be expressed in Quality Adjusted Life Years (QALYs), derived from utility scores, and used to determine the incremental cost-effectiveness ratio (ICER) expressed as the incremental costs per QALY. Given this study only uses routinely collected observational data, utility scores will be obtained by performing a mapping study to find EQ-5D-3L scores using the available HoNOS (see above). Costs and resources used included in both the CEA and CUA can be organized amongst two main categories:

1. Healthcare costs and resources:
  - Consumption and costs of healthcare resources related to the FACT+DSS or CAU teams. Upon consent, use of services will be requested for each participant by the involved healthcare providers and insurers. To observe differences in the units of services used by patients per service type, treatment groups will be made comparable using statistical analyses.
    - Direct time spent on patient care. Regular FACT team members are required to register direct and indirect time spent on each patient. To reduce the administrative burden of team members and increase time available for patient care, the Dutch Health Authorities exempted the mandatory registration of indirect time spent on a patient and simplified the registration of direct time for the duration of the study.
  - Healthcare costs for insured healthcare unrelated to FACT+DSS or CAU will be informed by the involved health insurance agency.
2. Other costs and resources:
  - Consumption and costs of social support services financed by the Social Support Act. e.g. household support, coaching, arranged housing, day activity centres, informed by the municipalities. Upon consent, use of services and costs (when available) will be requested for each participant. When applicable, patient out-of-pocket expenses will be included.
  - Consumption and costs of social security services financed by the Participation Act, e.g. social assistance benefits and employment re-integration services, informed by the municipalities. Upon consent, use of services and benefits will be requested for each participant.

Data regarding these categories will be collected in two ways 1) using individual patient data (IPD) of participants who have provided their informed consent, and 2) anonymous aggregated data for SMI patients within the study regions. Here, the aggregated data is expected to provide effect estimates, although less precise, that suffer less from selection bias that may occur during the process of informed consent collection. For example because patients with an overall worse state of mental health might be more unlikely, or unable, to provide consent. Aggregated data will be requested from the involved organizations using averages and deciles based on selection criteria identified from the IPD of participants. The use of aggregated data will increase our understanding of the impact of FACT+DSS teams. Resource use, costs and health-related outcome measures are collected starting ~ 1.5 year prior to the start, and until the end of the two-year study. Incremental differences between the CAU and FACT+DSS teams will be determined for costs, resource use and effects. The time horizon for the CUA and CEA will be 24 months, equivalent to the duration of the study.

### Process evaluation

A summative process and implementation evaluation will be performed to assess 1) if the FACT+DSS teams have been implemented as designed and have achieved their predefined goals, and 2) how the implementation process was experienced by its team members and how they evaluate the quality of care delivered, including its perceived impact on the target patient population. Finally, recommendations will be made for future implementation.

The process evaluation will be performed using the process evaluation framework by Saunders *et. al.* for health promotion programs [19]. This framework provides six steps to develop a process evaluation plan including the six elements necessary for a complete process evaluation: fidelity, dose delivered, dose received, reach, recruitment, and context. As suggested in the framework, process evaluation questions will be developed to address the six elements. To answer these questions, a mixed methods design will be used including both qualitative and quantitative data collection.

Quantitative data sources include administrative data, assessment of FACT fidelity scale scores, and the Questionnaire on the Experience and Evaluation of Work (QEEW), a survey for research on work, wellbeing and performance [10, 20]. The FACT fidelity scale consists of two parts where part A describes the team structure using a five point scale, and part B describes eight focus areas (making care flexible, personal domain, social domain, symptomatic domain, planning and monitoring at the individual client level, crisis and safety, social network collaboration, quality and innovation) that is scored using an eight point score. Scorings of both areas combined result in a maximum of 13 points to be obtained, where 6.6 or lower results in no certification, 6.7-7.4 in preliminary certification, and 7.5 or higher in receiving certification.

Qualitative data collection methods include document analysis (i.e. handbooks, implementation monitoring documents, project meeting minutes), walkalong notes, FACT fidelity scale assessment reports, and semi-structured interviews (i.e. with team members, project leaders, patient representative organisations). Document analysis will provide the initial foundation of data used to answer the process evaluation questions. Semi-structured interviews will be performed with team members, patient representatives and project leaders to supplement this data and fill information gaps. Patients will not be approached for interviews. All interviews will be audiotaped and transcribed.

## Analysis

### Sample size

For the current study a convenience sample will be used rather than conventional power analyses, where the aim will be to obtain consent from as many patients as possible. Given the pragmatic nature of the study, no additional data from patients is collected (i.e. besides data that is already routinely collected). As a result, the study population is by definition limited by the number of patients in each site. This limitation is partly mitigated by performing an economic evaluation as part of this study for which it is not costumery to perform power analyses, as for health-economic hypothesis testing one often requires extremely large sample sizes resulting from the large standard errors for costs. In addition, health-economic evaluations make use of probabilistic decision-making methods to draw conclusions on the relative cost-effectiveness of interventions and comparators. Moreover, aggregated data will be collected for all patients in each site, allowing for a validation of our analyses based on IPD with aggregated data.

### Effect analysis

Baseline data will be presented by presenting mean and standard deviations for continuous variables (or median and a percentile range when asymmetrical distributed) and by using counts and proportions for categorical variables. Differences in baseline patient characteristics between teams will be made comparable using inverse propensity scoring or entropy balancing methods [21]. Effects of the FACT+DSS teams on all predefined primary and secondary outcome measures will be evaluated using multi-level mixed model analysis with a random intercept for participant ID. For example, the fixed part of the model will have HoNOS scores as a function of condition (i.e. FACT+DSS teams or CAU), time, the interaction of condition x time. This example can also be written as:

$$\textrm{HoNOS}\sim \textrm{condition}+\textrm{time}+\textrm{condition}:\textrm{time}+\textrm{site}+\left(1|\textrm{id}\right)$$

Since there are only two implementation sites, these will not be modelled as a level in a multilevel mixed model, but as a series of indicator variables per site in the fixed part of the equation. Next, predictive marginal means will be computed and graphed in a margins plot to visualise the impact of condition on HoNOS over time. Because longitudinal mixed-modelling performs internal imputation, no other procedures for missing data are required [24]. All statistical analyses will be performed using the most recent version of R and R-Studio software available and the R package lme4 [25, 26].

### Economic analysis

Depending on the cost category and detail in the patient data received, either a gross-costing approach or micro-costing approach will be used. For example, the micro-costing approach may be applied when hours spent on specific resources or services are provided, for which appropriate unit costs can then be identified and reported.

Non-parametric bootstrapping (5000 times) will be used to assess differences in costs between the FACT+DSS teams and CAU teams. Non-parametric bootstrapping is a method based on random sampling with replacement based on the participants’ individual data [27]. Missing cost and outcome data will be imputed using single imputation using predictive mean matching nested in each bootstrap simulation to allow for intention-to-treat (ITT) analysis [28]. To correct for any baseline differences in costs and QALYs, seemingly unrelated regression equations will be applied to each bootstrap simulation to obtain incremental costs and incremental effects (i.e. QALYs or responder rate) [31]. Since the trial’s follow-up measurements will exceed 1 year discounting will be performed in line with the Dutch guidelines.

Results from the bootstrap simulations will be presented in both a cost-effectiveness plane and the cost-effectiveness acceptability curve (CEAC), where the probability of the FACT+DSS teams being cost-effective is given for a series of possible willingness-to-pay (WTP) thresholds [32. , 33]. Finally, the costs per patient will be combined with the total target patient population to estimate the budget impact of implementing the FACT+DSS teams.

### Process analysis

The main sources of data used in the process evaluation will be derived from document analysis and semi-structured interviews. Both interviews and documents will be coded and analysed using a directed content analysis approach [34]. Content analysis will be performed using the most recent version of the MAXQDA software available. Upon familiarization with the contents of the documents and transcribed interviews, open codes will be assigned to recurring themes and patterns using an integrated approach. To organize assigned codes, both a deductive and inductive approach will be used, categorizing codes in aggregate dimensions, and first order themes and second order concepts [35]. Here, the deductive code structure will describe the aggregate dimensions based on the elements of the process evaluation framework. First order themes and second order concepts will be created inductively based on the sensitizing concepts identified during coding [36]. All data will be analysed and coded by two researchers to optimize the consistency in this process.

## Discussion

This study aimed to present a research protocol describing the mixed-methods approach used to perform an effect evaluation, an economic evaluation, and a process and implementation evaluation of the integrated FACT+DSS teams that have been active throughout the two-year study period. In these intersectoral, multi-agency FACT+DSS teams the provision of health and social services is being further integrated which is expected to allow for a more holistic treatment approach, maximizing recovery and societal participation for those suffering from SMI, whilst also creating a more efficient environment for service provision.

Strengths of the research protocol presented here are its use of a mixed-method approach, combining quantitative and qualitative methods to evaluate the performance of the FACT+DSS teams on improving patient-related outcomes, staff and organization related outcomes, and economic outcomes. In addition to that, other important strengths are the use of real-world IPD supplemented with aggregated data, and its pragmatic design by collecting and combing routinely collected data from involved stakeholders to a single patient profile.

This study is potentially faced with several limitations and challenges that require additional attention. A number of limitations of this study are related to the pragmatic quasi-experimental design of the study and the limited number of teams and patients followed. First of all, uncertainty is expected to exist surrounding the availability and quality of the real-world patient data to be collected. As the current study depends on informed consent to obtain individual patient data for the (cost-) effectiveness analysis, challenges may arise during the process of patient inclusion, as frequently warned for in patients with SMI [37, 38]. In case of limited inclusion, the degree of uncertainty in the quantitative results of our analysis will increase.

Second, quasi-experimental studies are fundamentally more limited in their ability to identify causal relations due to the limitations of its design, such as its susceptibility to bias and confounding, and the increased incidence of data missingness [39]. However, we believe we can reduce these challenges by including aggregated data in our quantitative analyses, whilst also using the knowledge gained from our qualitative analysis as a foundation for any found effects (or the lack thereof).

Third, the objects of evaluation in the current study are limited to two FACT+DSS teams and three CAU teams based in two catchment areas, where, prior to the implementation, teams already showed important differences in baseline team composition, collaboration with network organizations in the region, and other small but relevant day-to-day activities of a FACT team. As such, this may challenge not only the identification of potential causal relations introduced by the implementation of FACT+DSS, but also its generalizability to other regions for future implementation. Overall, the generalizability of the findings could benefit from its pragmatic, quasi-experimental design, depending on the number of participants providing their informed consent and the quality of data provided by stakeholders.

Finally, a challenge our study faces relates to a national change in the system of how mental healthcare costs are charged per 2022. This new payment system is called the “care performance model” (in Dutch: Zorgprestatiemodel) where costs are now charged per service or contact (direct time spent) rather than a prescribed treatment plan or series of contacts. As a result, mental health service providers may increase their contacts and registration of direct time spent, and given one of the interests of this study is to identify if the integration of services affects the consumption of services, identifying this effect in this last half year of the study period may hold additional complexity. Also, in addition to this analytic challenge, the implementation of this new payment system is likely to affect the members of the FACT teams in their day-to-day operations, potentially posing them with additional (administrative) challenges that may affect the continued implementation and practice of the FACT+DSS approach. Therefore, attention to this change will also be included in the interviews for the process evaluation.

To the best of our knowledge, currently no other studies exist that implement the integration of mental health and social services to the same extent as the FACT+DSS teams. The FACT+DSS approach has been designed by an innovative stakeholder group in the Netherlands who aim to continue its development and rollout to other areas. Evidence and insights presented by this future study may support the decision-making process regarding further implementation of the integrated FACT+DSS approach, or similar initiatives in creating an integrated approach to mental healthcare.

## Data Availability

Data sharing is not applicable to this article as no datasets were generated or analysed during the current study.

## Abbreviations

SMI: Severe mental illness
FACT: Flexible Assertive Community Treatment
ACT: Assertive Community Treatment
DSST: District Social Service Teams
HoNOS: Health of the Nation Outcomes Scores
QoL: Quality of life
CAU: Care as usual
STROBE: Strengthening the Reporting of Observational Studies in Epidemiology
EIA: Employee Insurance Agency
EQ 5D-3L: EuroQol 5 Dimension 3 level
DSM-IV: Diagnostic and Statistical Manual of Mental Disorders Fourth Edition
CEA: Cost-effectiveness analysis
CUA: Cost-utility analysis
QALYs: Quality-adjusted life years
ICER: Incremental cost-effectiveness ratio
ITT: Intention-to-treat
WTP: Willingness-to-pay
CEAC: Cost-effectiveness acceptability curve
